# Strengthening oncology care in limited-resource settings through effective pharmacy quality improvement: insights from four major tertiary hospitals in Ghana

**DOI:** 10.3332/ecancer.2026.2129

**Published:** 2026-05-26

**Authors:** Joseph Daniels, Andrew Yaw Nyantakyi, Edwina Ayaaba Ayabilah, Kofi Adesi Kyei

**Affiliations:** 1 National Centre for Radiotherapy, Oncology, and Nuclear Medicine, Korle-Bu Teaching Hospital, Box KB 369, Accra, Ghana; 2 Department of Radiography, University of Ghana, Box KB 143, Legon, Accra, Ghana; 3 Department of Oncology, Cape Coast Teaching Hospital, Box CT 1363, Cape Coast, Ghana; a https://orcid.org/0000-0002-1466-150X; b https://orcid.org/0000-0003-0742-6007; c https://orcid.org/0009-0009-8399-4920; d https://orcid.org/0000-0003-3485-5368

**Keywords:** oncology care, pharmacy service, quality improvement, quality management, cancer pharmacotherapy

## Abstract

**Background:**

In resource-limited settings like Ghana, the growing burden of oncology care demands robust pharmaceutical quality improvement (QI) strategies to ensure safe and effective systemic cancer treatment. However, limited evidence exists on the implementation and impact of such strategies within pharmacy departments of tertiary healthcare facilities.

**Aim:**

The study assessed the effectiveness, impact and challenges associated with QI strategies and practices in the pharmacy departments of four major tertiary healthcare facilities in Ghana.

**Methods:**

A multi-centre cross-sectional study was conducted to assess QI practices in oncology care. A structured questionnaire and an observational checklist were used to collect data from 91 pharmacy staff who were recruited via stratified random sampling. Quantitative analysis was performed using STATA version 19 with descriptive and inferential statistics applied to evaluate the implementation and perceived effectiveness of QI strategies.

**Results:**

The mean age was 37.5 ± 9.9 years. Various QI strategies were widely implemented, including the use of technology (100%), standard operating procedures (92.3%) and staff training (83.5%). Key focus areas were inventory management, patient safety and reduction of medication error. While overall perceptions of QI effectiveness were high (mean = 4.92), training adequacy and resource allocation scored lower. Major challenges included theft, preferential treatment of patients and inventory issues. Regression analysis identified staff training (p = 0.026), patient-centered focus (p = 0.013) and responsiveness (p = 0.011) as significant predictors of perceived performance, underscoring the importance of these factors in enhancing pharmacy service quality.

**Conclusion:**

The study highlights the widespread adoption of QI strategies in Ghanaian tertiary hospital pharmacy departments and their perceived positive impact on oncology care delivery. Despite notable strengths in technology use and service orientation, gaps in staff training and resource allocation remain. Addressing these challenges through targeted investments and continuous professional development is essential to strengthen pharmaceutical services and ensure safer, more efficient cancer care in resource-limited settings.

## Introduction

Healthcare systems in low-resource settings are riddled with numerous challenges, particularly those that hamper the delivery of high-quality care for oncology patients [1, 2]. One of the crucial sectors contributing to the success of oncology care in healthcare facilities is the pharmacy department, where quality improvement (QI) strategies play a pivotal role in ensuring the availability, safety and efficacy of chemotherapeutic agents [3]. In pharmacy departments, QI strategies play a crucial role in optimising medication management, reducing errors and improving patient outcomes, especially for complex conditions like cancer [4]. In sub-Saharan Africa (SSA), as in many low-resource settings, the pharmacy departments of healthcare institutions are integral to the overall healthcare infrastructure, but they frequently face constraints in staffing, training, management of resources and limited access to advanced therapeutic agents and technologies [5]. Nevertheless, the ever-increasing burden of cancer in low-resource settings necessitates more robust and efficient healthcare delivery systems, with particular emphasis on the role of pharmacy services in oncology care [6].

Cancer is a leading cause of morbidity and mortality worldwide, and its incidence is rising in SSA, with breast, cervical, prostate and liver cancers being the most prevalent in Ghana [7]. This trend is exacerbated by demographic factors such as population growth, urbanisation and changing lifestyles, alongside limited access to early diagnosis, treatment and preventive services [8]. The situation in Ghana is reflective of broader trends across SSA, where cancer incidence rates are increasing due to a combination of environmental, genetic and lifestyle factors, but the healthcare systems remain ill-equipped to handle this growing burden [9]. As cancer management becomes more complex, improving the effectiveness of healthcare services in these settings, particularly in pharmacy departments, becomes increasingly critical to improving patient outcomes.

Pharmacy departments in tertiary healthcare facilities play a significant role in the management of oncology patients and must adopt structured QI frameworks to improve pharmacy services and enhance patient safety [10]. In Ghana, these departments provide not only the dispensing of medications but also clinical support in terms of drug therapy management, counseling and the provision of critical information regarding drug interactions, side effects and adherence to therapy regimens [11]. Unfortunately, these departments often lack standardised practices, effective training programs and adequate infrastructure to meet the growing demand for effective oncology care. QI strategies, which encompass efforts to enhance the processes, systems and structures within healthcare delivery, have been identified as a key approach to overcoming some of the challenges facing pharmacy services. These strategies include initiatives aimed at improving medication safety, reducing medication errors, enhancing communication among healthcare teams and ensuring optimal patient care through evidence-based practices. Given the increasing burden of oncology cases in Ghana, strengthening pharmacy services through effective QI measures is imperative to ensure the safe and timely delivery of chemotherapy and other essential systemic treatments [12]. Despite the recognition of QI strategies in healthcare systems worldwide, evidence on their implementation and effectiveness in SSA remains limited [13]. Ghana, like many other countries in the region, faces challenges such as inadequate healthcare infrastructure, limited access to essential medications and workforce shortages, which can negatively impact oncology care delivery [14]. There is an urgent need to explore how QI strategies contribute to improving the management of medications, patient safety and to identify potential barriers and facilitators to their successful implementation with regard to oncology care in limited-resource settings.

The aim of the study was to assess the effectiveness of QI strategies in the pharmacy departments of four major tertiary healthcare facilities in Ghana, and to explore the implications for oncology care in the subregion. The study also sought to identify areas where the adoption of further QI practices could improve pharmacy services in these settings. By identifying and evaluating effective QI strategies, this research informs policy development and healthcare practice in Ghana and other low-resource settings. Furthermore, by focusing on pharmacy departments, the study offers a unique perspective on how improvements in medication management can positively impact oncology care, contributing to evidence-based policy development.

## Methods

### Study design and setting

The research was a multi-centre cross-sectional quantitative study that was conducted at the pharmacy departments of four tertiary healthcare facilities in Ghana, all of which are central to the healthcare infrastructure of the country and serve as key referral centers for patients with cancer. These institutions were: Korle-Bu Teaching Hospital (KBTH), Cape Coast Teaching Hospital (CCTH), Greater Accra Regional Hospital (GARH) and Komfo Anokye Teaching Hospital (KATH). These pharmacy departments provide both oncological and non-oncological services to a diverse patient population. The cross-sectional nature of the study provided a snapshot of the current state of QI practices and their impact on oncology care, providing a robust basis for comparison across different healthcare settings. Notably, KBTH is the largest and most specialised public healthcare institution in Ghana. It is a regional and national referral center for oncology care, providing comprehensive cancer services, including surgery, chemotherapy, immunotherapy, targeted therapy, radiotherapy and palliative care services. Although smaller in size compared to KBTH, CCTH also plays an essential role in the delivery of cancer care, including the provision of medications for oncology patients. Located in the capital city of Accra, GARH is an ultramodern regional hospital serving a diverse population. Notably, KATH is the second-largest teaching hospital in Ghana with a pharmacy department that plays a critical role in the management of oncology patients, ensuring the availability and safe use of cancer drugs and supportive agents.

### Study population

The study involved pharmacists, pharmacy technicians, pharmacy assistants and support staff of the various pharmacy departments. All participants had knowledge and experience in medication dispensing, drug therapy management, patient-counseling and safety monitoring. The study included participants with varying levels of experience to capture a broad perspective on QI strategies.

### Study size and sampling strategy

The target sample size for the study was 90 participants. To account for potential non-response, questionnaires were administered to 100 eligible pharmacy staff across the four study sites, yielding a response rate of 91% (*n* = 91). Participants were recruited using a stratified random sampling technique. Strata were defined by professional cadre within each pharmacy department, namely licensed pharmacists, pharmacy technicians, pharmacy assistants and support staff. Because workforce composition varied across facilities and was subject to routine staffing changes, proportional stratification was based on contemporaneous departmental staff lists obtained at each site at the time of data collection rather than fixed centralised workforce totals. Within each stratum, participants were selected using simple random sampling. This approach ensured adequate representation of all professional categories involved in oncology-related pharmaceutical services while maintaining methodological transparency and minimising selection bias.

### Data collection

Data were collected using a structured questionnaire and an observational checklist. The questionnaire was administered in paper-based format, supervised by trained research assistants to ensure completeness and accuracy of responses. The questionnaire was designed to assess the implementation of QI strategies, their perceived effectiveness and impact on oncology-related pharmaceutical services. The observational checklist was used by the research team to validate self-reported QI practices, reduce social desirability bias and confirm the presence of standard operating procedures (SOPs) and inventory systems. It was administered by trained research assistants during site visits.

A pilot study was conducted at KBTH, to assess the clarity, flow and feasibility of the questionnaire. Only a small subset of staff who did not participate in the main survey were involved in the pilot, and no pilot data were included in the final analysis. Conducting the pilot at this facility allowed refinement of the survey instrument while ensuring that prior exposure did not influence responses in the main study.

### Data analysis

Quantitative data were analysed using STATA (StataCorp. 2025. Stata Statistical Software: Release 19. College Station, TX: StataCorp LLC.). Descriptive statistics, including means with standard deviations (SD), frequencies and percentages, were calculated to summarise participant characteristics and responses. Multiple linear regression analysis was conducted to assess the association between various QI practices and the perceived performance of the pharmacy departments, with ‘zero tolerance for mediocrity in the department’ as the dependent variable and QI practices (staff training, patient-centered focus, readiness to help patients, continuous service improvement and open-door policy) as independent variables. Statistical significance was set at *p* < 0.05.

### Ethical considerations

Ethical approval was obtained from the institutional review board prior to the commencement of the study. Additionally, written informed consent was obtained from all participants before data collection. Anonymity and confidentiality were maintained by assigning unique identifiers to each participant, and all data were securely stored and analysed anonymously.

## Results

In total, there were 57.1% male and 42.9% female participants with a mean age of 37.5 ± 9.9 years, ranging from 22 to 58 years, as summarised in Table 1. Approximately equal proportions of participants were recruited from each teaching hospital (KBTH = 26.4%, CCTH = 24.2%, GARH = 24.2% and KATH = 25.2%). Attainment of a Bachelor’s degree was the highest level of education for a considerable majority of the participants (67%). In all, 9.9% were licensed pharmacists, while 19.8% were pharmacy technicians. Also, 45.1% were pharmacy assistants, while 25.2% were pharmacy support staff. Most of the participants (59.3%) had been working at their respective pharmacy departments for 2 to 5 years, whereas 9.9% had > 5 years of working experience.

There was a strong consensus on the implementation and effectiveness of QI practices in the pharmacy departments as summarised in Table 2. The grand mean of 4.92 suggests that, on average, respondents strongly agreed that these practices were well established and positively impacted departmental operations. *‘Customer focus and quality are the driving forces behind our day-to-day operations’* (mean = 4.75) and *‘there is an open-door policy to encourage staff to air grievances’* (mean = 4.73) further highlight a strong emphasis on patient-centered service and staff engagement. Additionally, the high score for *‘QI strategies are reviewed and/or updated regularly’* (mean = 4.67) suggests that continuous improvement has been prioritised, ensuring that policies remain relevant and effective. However, the lower mean score for *‘every member of the department has been adequately trained’* (mean = 3.89) suggests some level of dissatisfaction or insufficiency in training efforts. Another area of relative concern was *‘adequate resources have been provided to support QI implementation’* (mean = 3.98).

Table 3 provides an overview of the QI **strategies** implemented, highlighting key focus areas and their prevalence. Among the reported strategies, the use of technology, such as Electronic Health Records (EHR) and barcode scanners, was the most widely implemented (100%), suggesting a strong reliance on digital tools to enhance efficiency, accuracy and patient safety. Additionally, *‘standard operating protocols were widely utilized’* (92.3%), emphasising strong reliance on structured protocols to improve workflow and reduce errors. Regular patient co﻿unseling and education programs, as well as the implementation of pharmacovigilance systems, were each reported by 75.8% of the respondents. Other frequently implemented strategies included efficient inventory management systems (85.7%) and staff training and continuous professional development (83.5%).

The top priorities for QI initiatives were *‘improving inventory management’* (85.7%), *‘enhancing patient safety’* (83.5%) and *‘increasing efficiency in dispensing’* (80.2%). *‘Reducing medication errors’* (78.1%) was also a major focal point, reflecting a strong emphasis on ensuring safe medication practices. *‘Increasing efficiency in dispensing’* (80.2%), highlighted efforts to minimise delays and streamline pharmacy workflows to ensure timely access to medications for patients. Also, 75.8% of the respondents alluded to *‘other’* QI focus areas, such as drug stewardship programs, cost-reduction efforts and workflow optimisation strategies. Overall, the findings demonstrate a balanced approach to QI, with a focus on both operational efficiency (inventory management, dispensing) and patient safety (reducing medication errors).

Table 4 summarises key challenges associated with implementing QI strategies. The grand mean of 3.53, suggests that respondents generally recognised these challenges as significant. Among the listed challenges, *‘theft and pilfering’* was the most pressing issue, with a mean score of 4.78, followed by *‘the preferential treatment of certain customers’* with a mean score of 4.29. Other notable concerns included wrong dispensing of drugs (3.97), poor inventory tracking (3.76) and trivialisation of customer complaints (3.82). Conversely, challenges such as lack of leadership support (2.35), inadequate number of trained staff (2.95) and inadequate drug storage facilities (2.30) received lower scores (lower than the grand mean of 3.53).

Table 5 summarises the perceived impact of various QI practices on the performance of the pharmacy departments. The dependent variable in the model was *‘there is zero tolerance for mediocrity in this department.’* The analysis was based on unstandardised coefficients (*B*), SD, *t*-values and *p*-values. The constant had a coefficient of −0.029 with a high *p*-value (0.910), suggesting that without the influence of the independent variables, the dependent variable was not significantly explained. Three independent variables had statistically significant effects on the perception of the performance of the pharmacy departments. *‘Training of department members’* (*B* = 0.596, *p* = 0.026) had the highest coefficient, indicating that adequate training of staff had the strongest impact on the perception of zero tolerance for mediocrity. Also, *‘customer focus and quality service’* (*B* = 0.465, *p* = 0.013) and *‘readiness to help customers’* (*B* = 0.351,* p* = 0.011) were significant. However, *‘continuous service improvement and customer satisfaction’* (*B* = 0.334, *p* = 0.225) and ‘open-door policy for staff grievances’ (*B* = 0.114, *p* = 0.435) were not statistically significant.

## Discussion

This multi-centre study provides important insights into the implementation and perceived impact of QI strategies within pharmacy departments of major tertiary hospitals in Ghana, with direct implications for oncology care delivery in resource-limited settings. Overall, the results demonstrate that QI practices are widely embedded in routine pharmacy operations, particularly in areas related to technology, staff training, inventory management and patient safety. While this is a positive outcome, it is also essential to contextualise these results within the scope of oncology care, where pharmacy services are crucial for the safe, effective and timely administration of chemotherapy and supportive therapies [15]. Medication errors, stock-outs and delays in dispensing can have profound consequences for patient outcomes.

In oncology care, focus on patient-centered service is particularly critical, as patients with cancer require personalised, timely and precise medication management. Effective communication and patient-centered care reduce medication errors, improve adherence to treatment regimens and enhance overall patient experience [4]. Furthermore, fostering an open-door policy allows pharmacists and staff to report workflow challenges, medication safety concerns or logistical bottlenecks that may impact oncology patients’ access to life-saving treatments.

The all-round implementation of digital tools indicates a well-established technological infrastructure supporting healthcare operations. The strong emphasis on technology adoption, including EHR and barcode systems, suggests a growing institutional commitment to modernising pharmacy workflows. In oncology settings, such technologies reduce prescribing and dispensing errors, improve traceability of cytotoxic medications and enhance pharmacovigilance. This is particularly relevant in Ghana, where increasing cancer incidence is placing growing pressure on already constrained health systems. However, the effectiveness of technological solutions is contingent upon adequate training and supportive infrastructure, areas identified in this study as requiring further strengthening.

The high adoption of inventory management practices highlights the importance of maintaining adequate stock levels, minimising wastage and ensuring the availability of essential medications. While slightly lower than the adoption rates of other strategies, the implementation of patient counseling & education programs, reported by 75.8%, suggests a significant effort toward patient-centered care. The focus on improving inventory management aligned with the high adoption of inventory management systems, indicating that the pharmacy departments place significant emphasis on optimising medication stock control and reducing shortages or overstocking. These priorities also suggest that facilities were actively working to prevent adverse drug events, whether through better prescription verification processes, improved labeling or adherence to SOPs.

The regular review and/or update of QI strategies signifies institutional commitment to continuous improvement. In the context of oncology care, this is essential given the rapid advancements in cancer treatment protocols, new drug approvals and evolving safety standards [12]. Regular updates to pharmacy protocols ensure that pharmacists and healthcare professionals remain current with best practices, reducing the risk of suboptimal treatment plans. Furthermore, continuous QI initiatives enhance pharmacovigilance efforts, ensuring that adverse drug reactions, particularly those associated with chemotherapy, are monitored and managed effectively [13].

The findings highlight several areas where structural and operational changes within pharmacy departments could enhance oncology service delivery. Key priorities include the establishment of dedicated oncology pharmacy units, with clearly defined roles for pharmacists, pharmacy technicians and assistants to optimise workflow and accountability. Implementing standardised protocols for inventory management, drug dispensing and patient counseling can minimise errors and improve efficiency.

One of the most critical findings relates to human resource capacity. Training adequacy emerged as the strongest predictor of perceived departmental performance, underscoring the central role of workforce development in sustaining QI. Effective oncology pharmacotherapy requires specialised competencies, including dose calculations, management of adverse drug interactions, dose modifications, safe handling of cytotoxic agents and patient-counselling [14]. Given that oncology pharmacy services involve specialised medications and infrastructure, addressing resource constraints could be particularly critical for sustaining QIs. Insufficient training could lead to medication errors, suboptimal dosing or improper handling of chemotherapy drugs, jeopardising patient safety [16]. Moreover, inadequate resource allocation may limit the availability of essential oncology medications, storage facilities and protective equipment necessary for the safe preparation of chemotherapy [17]. Resource constraints can also hinder the implementation of technology-driven QI strategies, such as computerised physician order entry and automated dispensing systems, which have been shown to improve medication safety in oncology settings [10]. The limited number of dedicated oncology pharmacists, underscores a critical workforce gap in the country’s cancer care system. Addressing these gaps through targeted investments in workforce development and infrastructure could significantly enhance the role of pharmacy departments in delivering high-quality oncology care.

Addressing the identified workforce gaps in oncology pharmacy requires targeted training and capacity-building initiatives. Training can be delivered through sponsored fellowship programs offered by the Ghana College of Pharmacists with a focus on oncology, complemented by structured continuing professional development programs, both hands-on and electronic, organised by the Pharmacy Council of Ghana. Universities and teaching hospitals can provide in-service training and mentorship opportunities, while the West African Postgraduate College of Pharmacists can facilitate regionally standardised postgraduate oncology programs. Additionally, international partnerships and twinning initiatives can offer expertise, mentorship and exposure to best practices in oncology pharmacy, strengthening the skills and competencies of Ghana’s oncology pharmacy workforce and improving the quality of cancer care delivery.

The study also reveals governance and ethical challenges, notably theft, pilfering and preferential treatment of patients. These issues threaten medication availability, equity and public trust. Addressing them requires both institutional and national responses. At the hospital level, Drug and Therapeutics

Committees, Audit Committees and strengthened internal control systems can improve accountability and inventory oversight. At the national level, collaboration with anti-corruption and regulatory bodies such as the Ghana Health Service, Ministry of Health, Pharmacy Counsel of Ghana, Economic and Organised Crime Office and Office of the Special Prosecutor may help address systemic vulnerabilities within pharmaceutical supply chains. Transparent procurement processes, routine audits and whistle-blower protections are essential components of sustainable reform.

The role of pharmacists within multidisciplinary oncology teams (MDTs) warrants particular attention. Notably, MDT-based cancer care is widely recognised as a gold standard for improving clinical outcomes, treatment coordination and patient safety. While MDTs exist within tertiary healthcare facilities in Ghana, pharmacist participation is inconsistent. Formal integration of pharmacists into MDTs would strengthen medication decision-making, optimise chemotherapy regimens and enhance monitoring of treatment-related toxicities. Therefore, MDT participation should be recognised as both a clinical and QI intervention.

Overall, the results suggest that while foundational QI structures are in place within Ghanaian tertiary hospital pharmacies, further progress will depend on targeted investments in workforce specialisation, governance mechanisms and oncology-specific pharmacy infrastructure. Strengthening these areas will be essential to ensuring safe, equitable and effective cancer pharmacotherapy in Ghana and similar resource-limited settings.

## Limitations

The cross-sectional design limits the ability to establish causality between QI strategies and observed outcomes in oncology pharmaceutical care. While associations were identified, longitudinal or experimental studies would be necessary to determine causal relationships. Second, the study was limited to four tertiary healthcare facilities, which, although major centers in Ghana, may not fully represent the experiences of pharmacy departments in secondary or primary care settings or in private institutions. This limits the generalisability of the findings to the broader healthcare landscape. Data were collected using self-administered questionnaires, which were subject to social desirability and recall biases. Participants may have overreported positive practices or underreported challenges to align with perceived expectations. Although an observational checklist was used to validate some responses, the potential for reporting bias remains. The use of the term ‘customer’ in the survey instrument may differ from conventional clinical terminology and could affect interpretation, as it captures patients, caregivers and relatives.

Chemotherapy reconstitution is a core function in oncology pharmacy practice that was not fully captured in the QI framework assessed in this study. The omission of this domain represents a limitation of the current study but also highlights the need for more comprehensive oncology-specific QI tools in future research. Furthermore, the institutional infrastructure necessary to support comprehensive QI interventions in oncology pharmacy practice was not fully evaluated in this study.

Only 9.9% of participants were licensed pharmacists, while pharmacy assistants constituted the largest proportion of respondents. Although this reflects the real-world workforce composition in Ghanaian tertiary hospitals, it may influence perceptions of QI effectiveness and limit conclusions specific to specialist oncology pharmacy practice.

## Conclusion

The results underscore a strong commitment to QI strategies within the pharmacy departments of tertiary healthcare facilities in Ghana, with significant positive implications for oncology care. However, challenges related to training and resource allocation must be addressed to ensure the highest standards of cancer treatment and medication safety. By investing in continuous professional development, enhancing pharmacovigilance systems and improving infrastructure, healthcare facilities can further optimise pharmacy services, ultimately improving outcomes for oncology patients. These healthcare facilities are not only working to optimise workflow efficiency but are also prioritising patient well-being. Staff training, patient-centered service readiness and a focus on quality are key drivers of the performance of pharmacy departments.

## Conflicts of interest

The authors declare no competing interest.

## Funding

This study did not receive any specific funding support from funding agencies in the public, commercial or not-for-profit sectors.

## Data availability

The data used to support the findings of this study are available from the corresponding author upon reasonable request.

## Figures and Tables

**Table 1.** Baseline characteristics of study participants (N = 91).

Characteristics	Variables	Frequency (n)	Percentage (%)
Healthcare facility	KBTH	24	26.4
	CCTH	22	24.2
	GARH	22	24.2
	KATH	23	25.2
Role in the pharmacy department	Pharmacist	9	9.9
	Pharmacy technician	18	19.8
	Pharmacy assistant	41	45.1
	Support staff	23	25.2
Number of years of working experience	< 2	28	30.8
	2–5	54	59.3
	> 5	9	9.9
KBTH: Korle-Bu Teaching Hospital, CCTH: Cape Coast Teaching Hospital, GARH: Greater Accra Regional Hospital, KATH: Komfo Anokye Teaching Hospital			

**Table 2.** QI practices (N = 91).

Characteristics		SD (1)	D (2)	U (3)	A (4)	SA (5)	Mean
Continuous improvement of services to enhance customer satisfaction	1		1	3	15	71	4.69
Training of members of the department	0		12	21	23	35	3.89
Adoption of an open-door policy to enable staff to air grievances, for prompt redress	0		2	3	13	73	4.73
Willingness to help customers* and the readiness to respond to customer requests	0		0	4	23	64	4.66
Customer focus and quality are the driving forces behind our day-to-day operations	1		1	2	12	75	4.75
Implementation of several simultaneous QI strategies	0		0	0	7	84	4.92
Frequent review/renewal of QI strategies	0		0	6	18	67	4.67
Allocation of adequate resources to support the implementation of QI strategies	6		7	10	28	40	3.98
QI: Quality improvement﻿, SD: strongly disagree, D: disagree, U: uncertain, A: agree, SA: strongly agree. *The original QI questionnaire employs the term ‘customer’ to describe service users. In the context of pharmacy services, ‘customers’ encompass not only patients but also caregivers, relatives and other individuals who interact with the pharmacy. Retaining this terminology ensures fidelity to the validated instrument and preserves the intended meaning of the survey items, which are designed to assess service delivery, user satisfaction and workflow efficiency. While the term ‘customer’ has been replaced with ‘patient’ in narrative sections to align with conventional clinical language, the original term is maintained in tables and direct survey items to accurately reflect the scope of service evaluation.							

**Table 3.** QI strategies implemented (N = 91).

Characteristics	Frequency (n)	Percentage (%)
**QI strategy**		
Updated SOP	84	92.3
Staff training and continuous professional development	76	83.5
Efficient inventory management systems	78	85.7
Regular patient counseling and education programs	69	75.8
Use of technology (e.g., EHR and barcode scanners)	91	100
Implementation of pharmacovigilance systems	69	75.8
**Primary focus of QI initiatives**		
Reducing medication errors	71	78.1
Improving inventory management	78	85.7
Enhancing patient safety	76	83.5
Increasing efficiency in dispensing	73	80.2
*Others	69	75.8
EHR: electronic health records. *Others included drug stewardship programs, cost-reduction efforts and workflow optimisation strategies. QI: quality improvement		

**Table 4.** Challenges associated with the implementation of QI practices and strategies.

Characteristics	SD (1)	D (2)	U (3)	A (4)	SA (5)	Mean
Preferential treatment of some customers above others	6	5	7	12	61	4.29
Theft and pilfering	0	0	0	20	71	4.78
Wrong dispensing of drugs	9	6	5	30	41	3.97
Poor inventory tracking	10	8	12	25	36	3.76
Trivialisation of customer complaints and feedback	5	12	10	31	33	3.82
High customer complaints	15	19	15	19	23	3.18
Insubordination	5	4	12	30	40	4.05
﻿Inadequate funding	6	7	10	28	40	3.98
Lack of leadership support	26	33	14	10	8	2.35
Inadequate number of staff	11	21	30	20	9	2.95
Insufficient training	0	12	21	23	35	3.89
Resistance to change	9	11	11	26	34	3.71
Staff mediocrity & indifference	25	33	15	10	8	2.37
Inadequate drug storage facilities	26	36	12	10	7	2.3
SD: strongly disagree, D: disagree, U: uncertain, A: agree, SA: strongly agree						

**Table 5.** The perceived impact of QI practices on the performance of the pharmacy departments.

Model	Unstandardized coefficients		t	p - value
	B	SD		
(Constant)	−0.029	0.243	−0.116	0.910
The continuous improvement of services has greatly enhanced customer satisfaction	0.334	0.091	1.973	0.225
Every member of the department has been adequately trained	0.596	0.065	2.691	0.026
There is an open-door policy to encourage staff to air out their grievances for prompt redress	0.114	0.055	1.733	0.435
The department is well known for the willingness to help customers and readiness to respond to customer requests	0.351	0.069	2.445	0.011
Customer focus and quality service are the driving forces behind the day-to-day operations of the department	0.465	0.093	2.112	0.013
